# Supplementary material for: Agile Free‐Form Signal Filtering and Routing with a Chaotic‐Cavity‐Backed Non‐Local Programmable Metasurface
Source: Adv Sci (Weinh). 2025 Feb 25;12(16):2500796. doi: 10.1002/advs.202500796 (PMC12021040; doi:10.1002/advs.202500796)
Supplement: Supplementary file 1 — Supporting Information [file ADVS-12-2500796-s001.pdf]

ADVANCED SCIENCE
Open Access

Supporting Information

for *Adv. Sci.*, DOI 10.1002/advs.202500796

Agile Free-Form Signal Filtering and Routing with a Chaotic-Cavity-Backed Non-Local Programmable Metasurface

*Fabian T. Faul, Laurent Cronier, Ali Alhulaymi, A. Douglas Stone and Philipp del Hougne**

Supporting Information:

Agile Free-Form Signal Filtering and Routing with a Chaotic-Cavity-Backed Non-Local Programmable Metasurface

Fabian T. Faul[1], Laurent Cronier[1], Ali Alhulaymi[2], A. Douglas Stone[2], Philipp del Hougne[1]*

[1] Univ Rennes, CNRS, IETR-UMR 6164, F-35000 Rennes, France

[2] Department of Applied Physics, Yale University, New Haven, CT 06520, USA

* Correspondence: philipp.del-hougne@univ-rennes.fr

Table of Contents

S1. Raw Scattering Coefficients Measured for Three Random Metasurface Configurations.........2

S2. Frequency-Dependence of Occurrence Rates...3

S3. Comparison With Literature For Reconfigurable Multi-Band Filtering...................................4

Supplementary References...5

S1. Raw Scattering Coefficients Measured for Three Random Metasurface Configurations

We display in Figure S1 below the raw scattering coefficient spectra measured for three random metasurface configurations. These spectra display a seemingly random frequency dependence of the scattering coefficients which originates from complex interference processes within the chaotic cavity and should not be mistaken for measurement noise. The dynamic range of our measurements exceeds 50 dB. Note also that due to energy conservation it is not possible that all transmission coefficients have simultaneously near-unity magnitudes at any given frequency; hence, the fact that the scattering coefficient magnitudes are below unity should not be mistaken to constitute an indicator of strong absorption.

In Figure 1b in our main text, we display an analysis of the dependence of these scattering coefficient spectra on the metasurface configuration. Specifically, based on 2040 random metasurface configurations, we display the maximum, minimum, average and standard deviation for each frequency point.

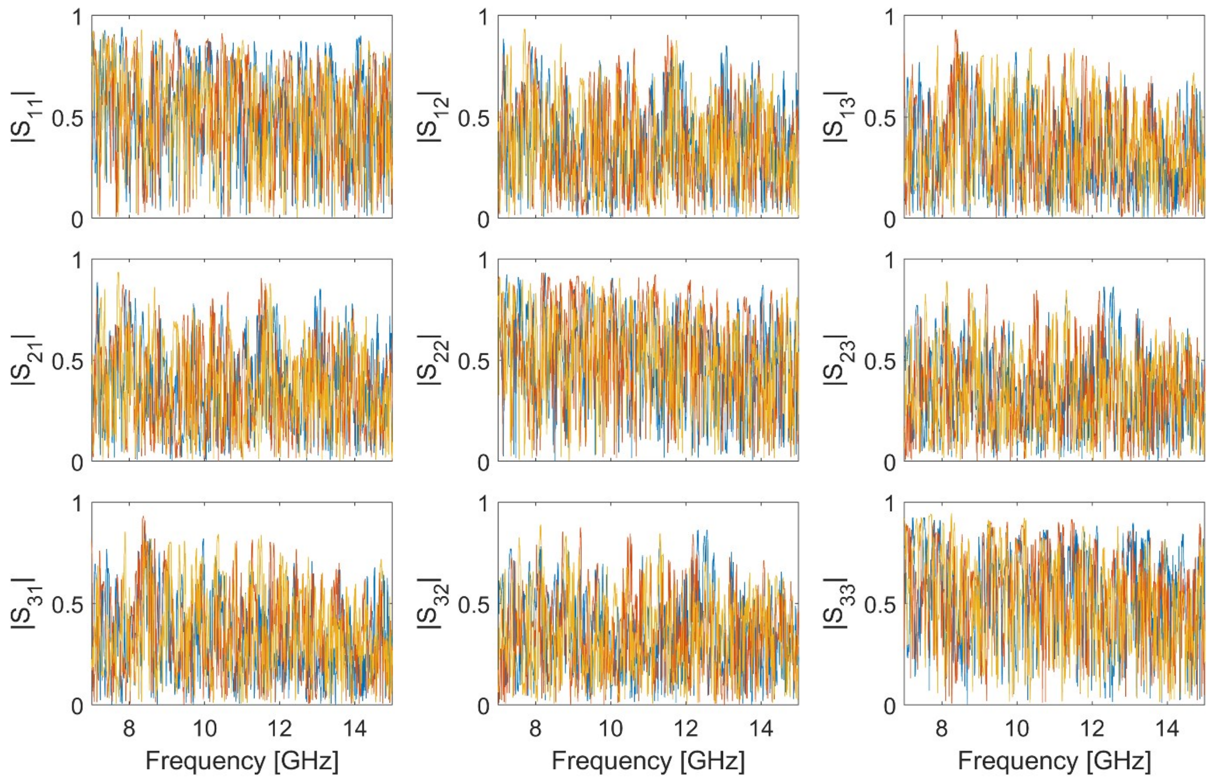

Figure S1. Raw scattering coefficients measured for three random metasurface configurations.

S2. Frequency-Dependence of Occurrence Rates

The occurrence rates displayed in Figure 2a in the main text are averaged over all frequency points within the considered interval from 7 GHz to 15 GHz. Because the absorption is not constant but slightly increases as a function of frequency, we provide a more fine-grained analysis here. Specifically, we divide the considered 8-GHz-wide interval into four 2-GHz-wide intervals and repeat the analysis for each of these four smaller intervals. The results are displayed in Figure S2 below.

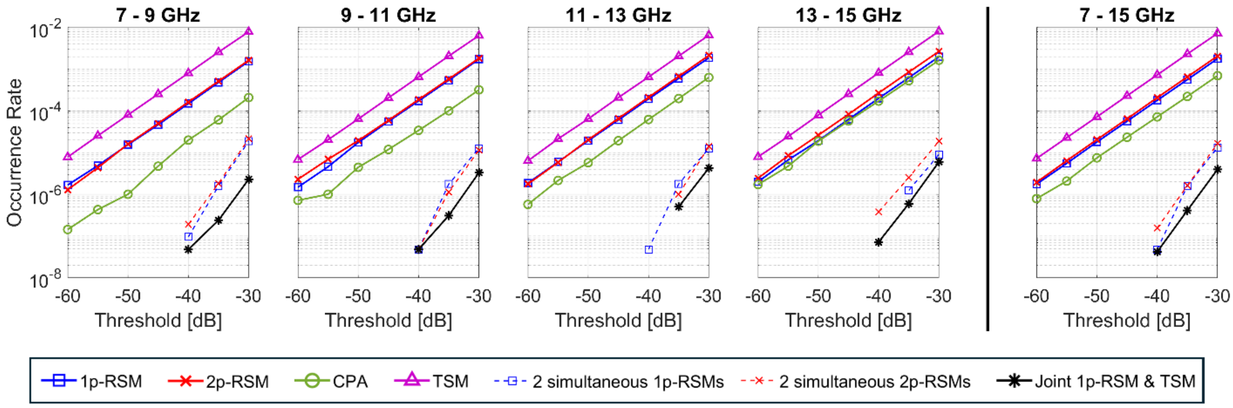

Figure S2. Frequency-dependence of occurrence rates. The occurrence rates as a function of threshold evaluated over four adjacent 2-GHz intervals are displayed on the left. For reference, the occurrence rates evaluated over the entire 8-GHz interval are reproduced on the right (identical to Figure 2a in the main text).

First, we note that the curves on the left-hand side in Figure S2 are slightly less smooth in the regime of low occurrence rates because they are based on four times fewer samples than Figure 2a (which is reproduced on the right-hand side in Figure S2).

Second, we note several interesting tendencies as the central frequency of the considered 2-GHz-wide interval is increased from 8 GHz via 10 GHz and 12 GHz to 14 GHz, alongside which the level of absorption increases. First, we observe that CPA becomes notably more likely. This makes sense because CPA is impossible in the limit of no loss such that increasing the absorption in the low-loss regime is expected to significantly increase the rate at which CPA can be observed. However, even in the 13-15 GHz interval, CPA does not have higher occurrence rates than other RSM types, indicating that overall the level of absorption is still not high – in contrast to the prototype in Ref.[S1]. Second, we observe that a slight difference between the occurrence rates of 1p-RSM and 2p-RSM arises as we increase the central frequency of the considered interval: 2p-RSMs become slightly more likely as the absorption level increases. This also makes sense, since absorption shifts the distributions of 1p-RSMs and 2p-RSMs slightly down in the complex plane: For a lossless system, the two distributions are expected to be complex conjugates of each other and thus equally distant from the real frequency axis, resulting in equal occurrence rates of 1p-RSMs and 2p-RSMs; meanwhile, for a system with a small level of absorption, the 2p-RSM distribution is slightly closer to the real axis and hence the 2p-RSM occurrence rate is slightly higher. At the same time, we observe that the TSM occurrence rates do not display any significant dependence on the absorption rate within the explored regimes.

S3. Comparison With Literature For Reconfigurable Multi-Band Filtering

In this section, we attempt to compare the performance of our prototype reported in Figure 6 with the state-of-the-art literature on reconfigurable multi-band filtering. However, it is important to note that this comparison is necessarily limited. We are not aware of any system offering a level of “free-form tunability” comparable to our system. Therefore, the present section compares only one functionality enabled by our prototype, namely reconfigurable multi-band filtering, with “best-in-class” devices that are only capable of reconfigurable multi-band filtering. In particular, none of the state-of-the-art devices mentioned here could in addition implement reflectionless reconfigurable signal routing. In addition, our work does not present a mature technology because it is focused on the physics underlying this fundamentally different approach to agile free-form filtering. Finally, our optimization criteria for multi-band reconfigurable filtering were not identical to those of the state-of-the-art devices mentioned here. Our optimization criteria solely related to maximizing transmission in specified passbands and minimizing transmission in specified stopbands. In line with the envisioned application to scenarios requiring the reception of weak band-agile signals in close spatial proximity to the emission of strong band-agile signals, the specified stopbands in our work were of comparable width to the passbands as opposed to assigning all frequency points outside the passbands to stopbands.

Reference	Ref.[S2]	Ref.[S3]	Ref.[S4]	Ref.[S5]	Fig. 6 of our work
**Tuning Mechanism**	Varactors	Varactors	Varactors	Trimmer Capacitors	Stepper Motors
**Reported Tunability**	Dual-Band with $1.48<f_1<1.80$ and $2.40<f_2<2.88$	Dual-Band with $0.77<f_1<1.02$ and $1.57<f_2<2.00$	Dual-Band with $1.15<f_1<1.72$ and $2.12<f_2<2.45$	Multi-Band between 0.8 GHz and 1.4 GHz	Multi-Band between 7 GHz and 15 GHz
**Stopband Suppression**	24 dB	Roughly 20 dB (read off plot).	Roughly 23 dB (read off plot).	Roughly 25 dB (read off plot).	At least 24 dB.
**Passband Attenuation (Insertion Loss)**	Between 2.0 dB and 4.4 dB.	Between 0.7 dB and 3.9 dB.	Between 1.7 dB and 5.2 dB.	Between 1.8 dB and 3 dB.	1 dB
**Reconfiguration Speed**	Not indicated.	Not indicated.	Not indicated.	Not indicated.	~ 1 second (not optimized)
**Energy Consumption for Reconfiguration**	Not indicated.	Not indicated.	Not indicated.	Not indicated.	~ 6J / element (not optimized)

Table S1. Comparison with representative literature for reconfigurable multi-band filtering.

The following important observations can be made as a result of this comparison:

- Existing demonstrations of reconfigurable multi-band filters exist in the 1 GHz regime. We consider one order of magnitude larger frequencies around 10 GHz. Attenuation increases with frequency. Nonetheless, the passband attenuation in our work is lower than in the literature examples cited above.
- Existing reconfigurable multi-band filters are often limited to dual-band operation.
- The fractional bandwidth over which the response can be tuned is roughly one octave in our work, which is comparable to the best-in-class result in the literature (Ref.[S5]).

Supplementary References

[S1] J. Sol, A. Alhulaymi, A. D. Stone, P. del Hougne, *Sci. Adv.* **2023**, *9*, eadf0323.

[S2] G. Chaudhary, Y. Jeong, J. Lim, *IEEE Trans. Microw. Theory Techn.* **2013**, *61*, 107.

[S3] X. Huang, L. Zhu, Q. Feng, Q. Xiang, D. Jia, *IEEE Trans. Microw. Theory Techn.* **2013**, *61*, 3200.

[S4] T. Yang, G. M. Rebeiz, *IEEE Trans. Microw. Theory Techn.* **2013**, *61*, 3613.

[S5] R. Gomez-Garcia, A. C. Guyette, *IEEE Trans. Microw. Theory Techn.* **2015**, *63*, 1294.
